# A recommendation of PHTALOX® for preventing infection and progression of COVID-19: a 1-year summarized update of scientific approaches

**DOI:** 10.3205/dgkh000406

**Published:** 2022-01-31

**Authors:** Bernardo da Fonseca Orcina, Verônica Caroline Brito Reia, Andréa Name Colado Simão, Audrey Alesandra Stinghen Garcia Lonni, Thais Maria Freire Fernandes, Marcelo Lupion Poleti, Fabiano Vieira Vilhena, Paulo Sérgio da Silva Santos

**Affiliations:** 1 University of Sao Paulo, Bauru School of Dentistry, Department of Surgery, Stomatology, Pathology and Radiology Bauru, Brazil; 2 State University of Londrina, Londrina, Brazil; 3 Department of Orthodontics, University of North Paraná (UNOPAR), Londrina, Brazil; 4 Federal Institute of Parana, Londrina, Brazil; 5 TRIALS – Oral Health & Technologies, Bauru, Brazil

## Letter to the editor

Dear editor,

The role of the oral cavity in the genesis, progression, and dissemination of COVID-19 has been revealed by breakthroughs in SARS-CoV-2 research [1]. As a result, in 2020, our research group presented a recommendation for PHTALOX^®^ mouthwash for reducing COVID-19 infection and progression [2]. On this occasion, we revealed the first clinical findings in patients with COVID-19 who utilized PHTALOX^®^, an antiviral phthalocyanine derivative (APD), in a gargle/rinse mouthwash protocol [3]. We updated the information with new insights on the usage of the APD method since PHTALOX^®^ was indicated against SARS-CoV-2 (Table 1 (Tab. 1)).

Hence, combined with immunizations, the activity of products containing PHTALOX^®^ may help prevent patients from transmitting SARS-CoV-2 and thus help prevent others from contracting COVID-19. Following this logic, additional findings from our study group underscore the positive impact of APD. In epidemiological research, the usage of products containing APD reduced virus dissemination and COVID-19 symptoms [4]. In a population-based study with a sample that used APD, a reduction (p<0.05) in the incidence of COVID-19 was observed compared to a control population without APD exposure [5]. Thus, while vaccines are the most important tool in combating the COVID-19 pandemic, they are not 100% effective in those vaccinated, and even after the initial dose, antibody production takes several days to mature, followed by reinforcement with additional doses [6], at which point gargle-and-rinse solutions may be an effective option.

As previously indicated, we emphasize how important it is for scientists and governments to evaluate the impact of APD policies in hospitals and the general community on SARS-COV-2 VL, hence reducing the virus's transmission and severity of COVID-19.

## Notes

### Competing interests

All authors submitted the ICMJE Form for Disclosure of Potential Conflicts of Interest. Dr. Vilhena reports personal fees from TRIALS Inc. while conducting the study. In addition, Dr. Vilhena has a patent pending. Dr. da Silva Santos reports grants from CNPq process nº. 309525/2018-7. The other authors claim no conflicts of interest.

### Acknowledgments

This study was financed in part by the Coordenação de Aperfeiçoamento de Pessoal de Nível Superior (CAPES), Brazil (Finance Code 001).

### ORCID-ID of the Authors

- Bernardo da Fonseca Orcina:
https://orcid.org/0000-0003-3367-483X
- Verônica Caroline Brito Reia:
https://orcid.org/0000-0003-1352-5474
- Andrea Name Colado Simão:
https://orcid.org/0000-0002-2073-6782
- Audrey Alesandra Stinghen Garcia Lonni:
https://orcid.org/0000-0001-6498-2806
- Thais Maria Freire Fernandes:
https://orcid.org/ 0000-0002-4368-8568
- Marcelo Lupion Poleti:
https://orcid.org/0000-0003-1904-5762
- Fabiano Vieira Vilhena:
  https://orcid.org/0000-0003-3840-3633
- Paulo Sérgio da Silva Santos:
https://orcid.org/0000-0002-0674-3759

## Figures and Tables

**Table 1:**
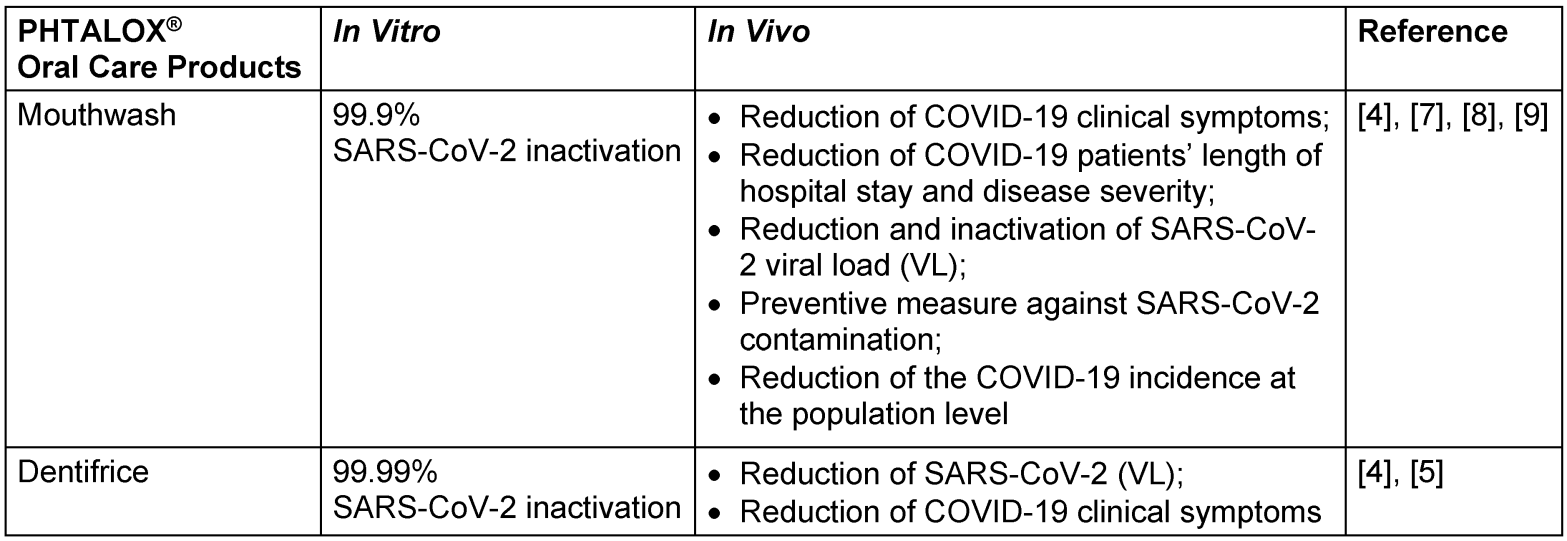
Summarized results for the use of PHTALOX^®^ against COVID-19
